# Occlusive thrombosis in arteries

**DOI:** 10.1063/1.5115554

**Published:** 2019-11-19

**Authors:** Dongjune Kim, Christopher Bresette, Zixiang Liu, David N. Ku

**Affiliations:** GWW School of Mechanical Engineering, Georgia Institute of Technology, Atlanta, Georgia 30332-0363, USA

## Abstract

Thrombus formation in major arteries is life threatening. In this review article, we discuss how an arterial thrombus can form under pathologically high shear stresses, with bonding rates estimated to be the fastest $K_{on}$ values in biochemistry. During occlusive thrombosis in arteries, the growth rate of the thrombus explodes to capture a billion platelets in about 10 min. Close to 100% of all platelets passing the thrombus are captured by long von Willebrand factor (vWF) strands that quickly form tethered nets. The nets grow in patches where shear stress is high, and the local concentration of vWF is elevated due to α-granule release by previously captured platelets. This rapidly formed thrombus has few red blood cells and so has a white appearance and is much stronger and more porous than clots formed through coagulation. Understanding and modeling the biophysics of this event can predict totally new approaches to prevent and treat heart attacks and strokes.

## I. WHITE CLOT FORMATION IN ARTERIES

**FIG. 1.**
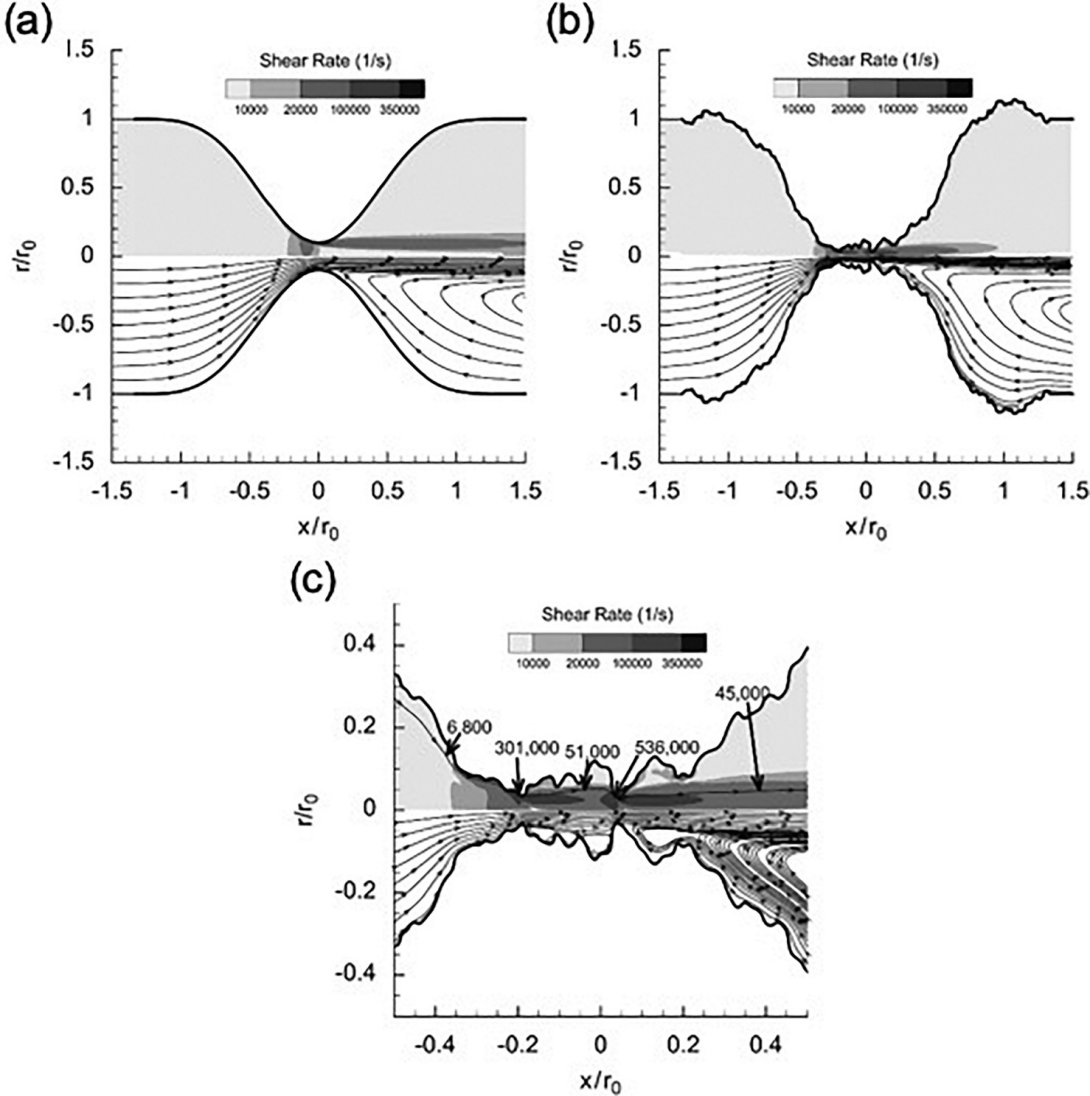
Flow patterns in a stenosis with a smooth surface (a), with a rough thrombus surface (b), and magnified view of throat (c). Note that the rough thrombus creates areas of extremely high and low shear rates at the surface. Reproduced with permission from D. L. Bark, Jr. and D. N. Ku, J. Biomech. **43**, 2970–2977 (2010). Copyright 2010 Elsevier Ltd.

Acute arterial thrombosis is a condition where a blood clot forms in an artery, stopping blood flow once the clot becomes occlusive. When thrombosis occurs in a carotid or coronary artery, it can result in ischemic stroke or myocardial infarction, two of the leading causes of death worldwide. The path to arterial thrombosis typically starts with atherosclerosis. The atherosclerotic plaque develops in the vessels over decades, creating a stenosis that narrows the lumen of the vessel and changes blood flow. In order for blood flow to be maintained, the velocity of the blood is increased, and pathologically high wall shear rates are created. Shear rates here can range from 5000 to 400 000 $s^{-1}$ (Fig. 1), much higher than the typical shear rates of <1000 $s^{-1}\mathrm{in}\;\mathrm{normal}\;\mathrm{arteries}.$^1,2^ After the plaque cap of the stenosis ruptures, the endothelial cell layer is disturbed and prothrombogenic collagen from the extracellular matrix is exposed. These events lead to the tethering and elongation of von Willebrand factor (vWF), platelet adhesion, and shear-induced platelet aggregation (SIPA), resulting in an occlusive blood clot formation and cessation of the blood flow.^3^

**FIG. 2.**
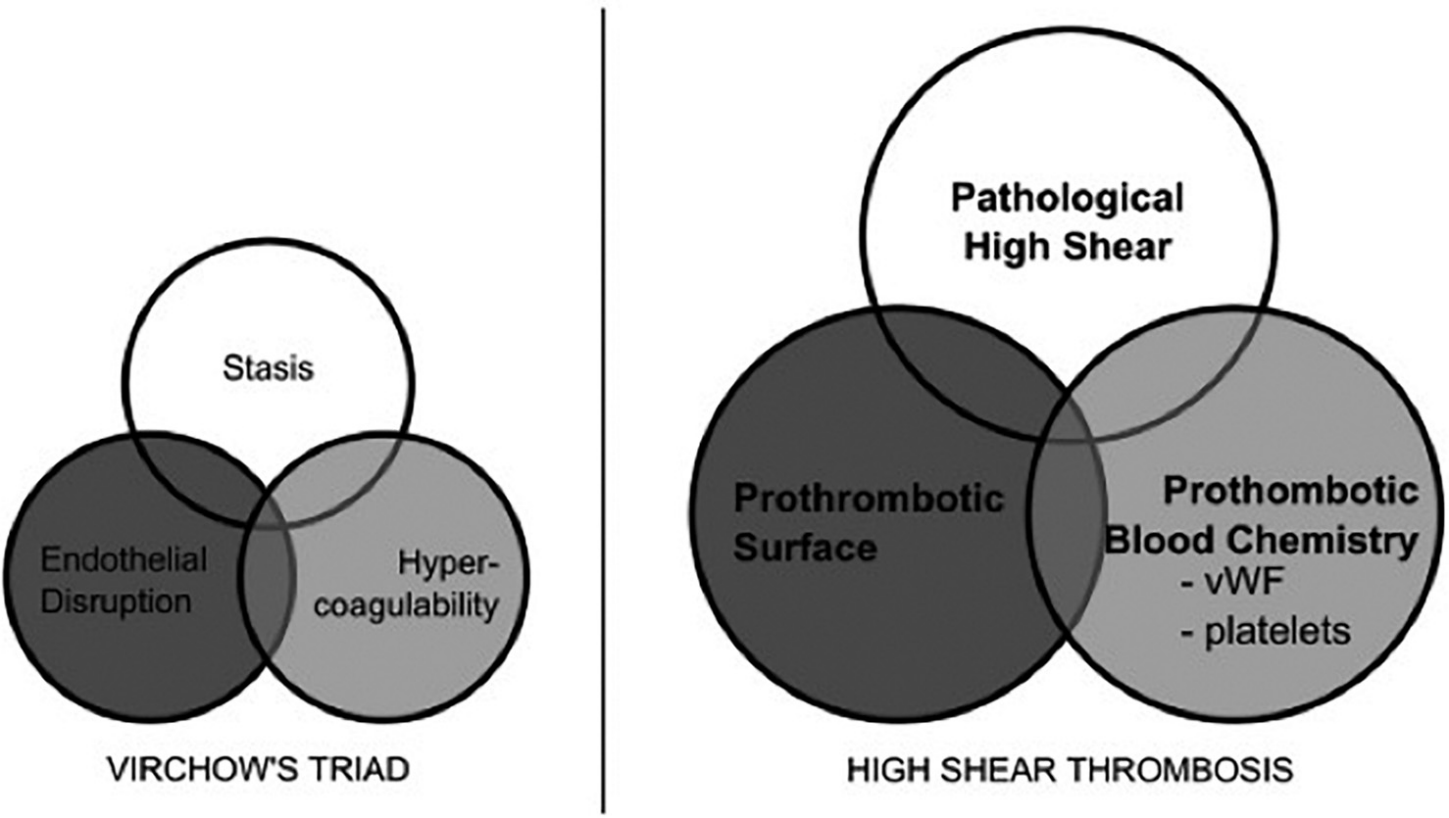
Virchow's triad and alternative triad for the high shear thrombosis. Reproduced with permission from Casa *et al.*, J. Vasc. Surg. **61**, 1068–1080 (2015). Copyright 2015 Elsevier Ltd.

The clots that form due to arterial thrombosis have a unique appearance compared to the typical blood clots formed through the coagulation cascade. Compared to the red coagulation clots, thrombi in arteries often appear light due to the relative absence of erythrocytes and prevalence of platelets. Because of this, they are commonly called white clots. Beyond appearances, the mechanism of the white clot formation is different from the classic Virchow's triad of coagulation-based red clotting (Fig. 2). Virchow's triad consists of endothelial disruption, stagnant blood flow, and hypercoagulability.^4^ Casa *et al.*^3^ suggest an alternative triad necessary for arterial thrombosis in contrast to Virchow's triad: (1) surface with collagen or other substrates to initially absorb vWF, (2) pathologically high shear for vWF elongation, and (3) platelets and vWF at sufficient concentrations (Fig. 2). There is little overlap between the two, and the only trait of Virchow's triad present for high shear arterial thrombosis *in vivo* is the endothelial disruption/prothrombotic surface. Regardless, several investigators have attempted to predict arterial thrombosis using Virchow's triad, measuring hemodynamic low shear to indicate stagnant blood flow and activated coagulation factor levels or activated platelets for hypercoagulability. However, there is plenty of evidence, suggesting using Virchow's triad is inappropriate for arterial thrombosis. Cadroy *et al.*^5^ showed white clots form at high shear rates, whereas red clots are created in low shear regions. Platelet activation, spreading, activation of coagulation factors, and thrombin generation can contribute to stabilization in the interior of a clot, but these processes occur too slowly to account for the rapid growth rates seen in the high shear environment of a stenotic artery.^2^ Circulating platelets have been found to activate in solution experimentally, according to a curve given by Hellums.^6^ However, circulating platelets pass through a stenosis in milliseconds, too quickly for activation on the Hellums graph. In fact, Ruggeri *et al.*^7^ found that platelets form aggregates without activation when shear rates exceed 10 000 $s^{-1}$ and soluble von Willebrand Factor (vWF) is present. Casa *et al.*^8^ later demonstrated that occlusive high shear thrombus does not form if the vWF concentration is decreased by 90% but can occur with a normal vWF concentration and a 10% platelet concentration. Thus, these findings have established the primacy of vWF in creating occlusive thrombi under arterial conditions.

The analysis of Wellings and Ku^9^ illustrates the amazing ability of platelets to stick on the upstream side of a stenosis further demonstrating the importance of vWF. To capture platelets during their extremely short transit times, vWF must bind platelets with theoretical $K_{on}$ rates from $10^{5}$ to $10^{9}\;M^{-1}\;s^{-1}$, which can be made possible through unfolding of vWF at a high concentration to expose an abundance of A1 domains, the platelet binding site on vWF. Recently, single molecule studies by Fu *et al.*^10^ confirmed that the $K_{on}$ rate of platelets to vWF A1 is greater than $10^{6}\;M^{-1}\;s^{-1}$, experimentally. A high concentration of vWF is achievable if previously captured platelets activate and locally release tethered vWF at a 50× plasma concentration from their α-granules.^9^ The high concentration of elongated vWF can then self-associate into tethered nets that encircle platelets to multivalently form thousands of GPIbα-A1 bonds and resist the high drag forces on mural platelets. Trapped and activated platelets also form integrin α_IIb_β_III_ (GPIIb/IIIa) bonds necessary for a stable clot and release FVIII to initiate coagulation, which, for arterial thrombosis, is a much slower secondary process. Thus, the biophysics of an occlusive thrombus must involve the superfast $K_{on}$ rate of the GPIbα-A1 bond with multivalent bonding favored by high local concentrations of vWF.^9^

## II. EXPLOSIVE PLATELET ACCUMULATION VIA vWF

During events like ischemic stroke or myocardial infarction, the formation of an occlusive white clot rapidly halts blood flow through the artery. Histology studies estimate white clots to be approximately 50%–80% platelets by volume.^11^ To occlude an artery with a diameter >4 mm, billions of platelets must be captured within a couple of hours.^12^ However, most previous studies have only focused on the initial adhesion and aggregation of up to thousands of platelets within the first 2 min of perfusion. Our studies ending in occlusion show that the initial attachment is relatively slow, even with high shear stresses, and is possibly more related to the initial adsorption of vWF to the collagen-coated surface.^9^ We and others have described the different phases of white clot growth under high shear, even in clots that do not go to full occlusion.^13^

The presence of collagen on a surface in our *in vitro* stenoses or in *in vivo* atherosclerotic stenoses following plaque cap rupture can initiate the formation of an occlusive clot. After the plaque cap rupture, marginated soluble vWF can bind to exposed fibrillar collagen type 1 or 3 through the A3 domain, mediated by the A2 domain.^14–16^ The adsorption rate of vWF onto the collagen-coated surface increases with the vWF molecular weight^17^ and shear rates above the threshold for unfolding the vWF molecule.^18^ For shear rates over 1000 $s^{-1}$, vWF becomes the dominant molecule that mediates platelet binding.^19^ Globular tethered vWF experiences high shear and elongates, thereby exposing an order of magnitude more A1 domains to capture thousands of platelets.^9^

vWF tethered to collagen surfaces arrests platelets in blood flow via GPIbα-A1 binding. The platelet adhesion rate is aided by platelet margination, where the hematocrit and shear rate can increase the platelet concentration near the vessel wall by 2.5 fold.^20^ GPIbα is a glycoprotein receptor on the membrane of unactivated platelets that can bind the vWF A1 domain, while the platelet is traversing the stenosis.^7^ Since the high shear environment applies a large hydrodynamic force on platelets, exceeding 10 000 pN, over 1000 GPIbα-A1 bonds with an individual strength of <100 pN are required to resist the hydrodynamic drag force and stop a platelet in flow.^21^

**FIG. 3.**
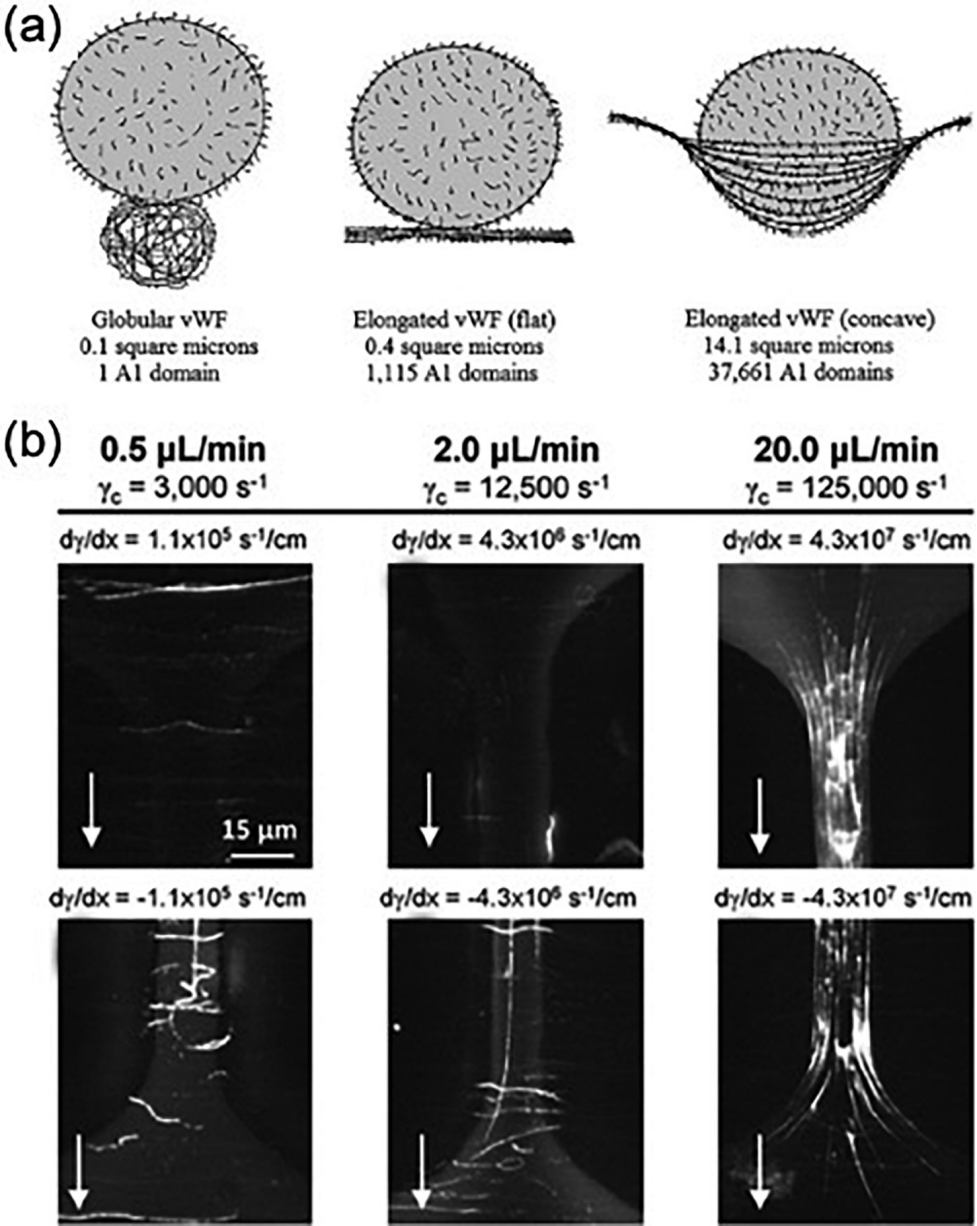
von Willebrand factor (vWF) nets. (a) vWF extends from a globular form to an elongated form at a high shear rate and can theoretically form intertwining nets to form many bonds to platelets, capturing them from the high shear flow. Reprinted with permission from P. J. Welling and D. N. Ku, “Mechanisms of platelet capture under very high shear,” Cardiovasc. Eng. Technol. **3**, 161–170 (2012). Copyright 2012 Springer Nature. (b) Experimental demonstration of the vWF nets at very high shear rates. The vWF strands are shown in white. Reproduced with permission from T. V. Colace and S. L. Diamond, Arterioscler., Thromb., Vasc. Biol. **33**, 105–113 (2013). Copyright 2013 American Heart Association, Inc.

Platelet adhesion and aggregation through GPIbα-A1 association are not stable on its own. After initial binding, platelets can activate in the clot. These activated platelets form more stable bonds with vWF via integrin α_IIb_β_III_-C1 binding and initiate the coagulation cascade through the release of procoagulant molecules from α-granules like adenosine 50-diphosphate, calcium ions, and inflammatory factors.^6,22^ Platelet vWF stored in the α-granule is released, causing larger structures to form and rapid platelet aggregation (Fig. 3).^23^ Capture of platelets on this reactive surface and subsequent platelet activation creates a positive-feedback loop that can result in billions of platelets aggregating at almost 10 times the rate of initial attachment to the thrombogenic surface.

## III. SUMMARIZING RPA AND PLATELET CAPTURE BY vWF

**FIG. 4.**
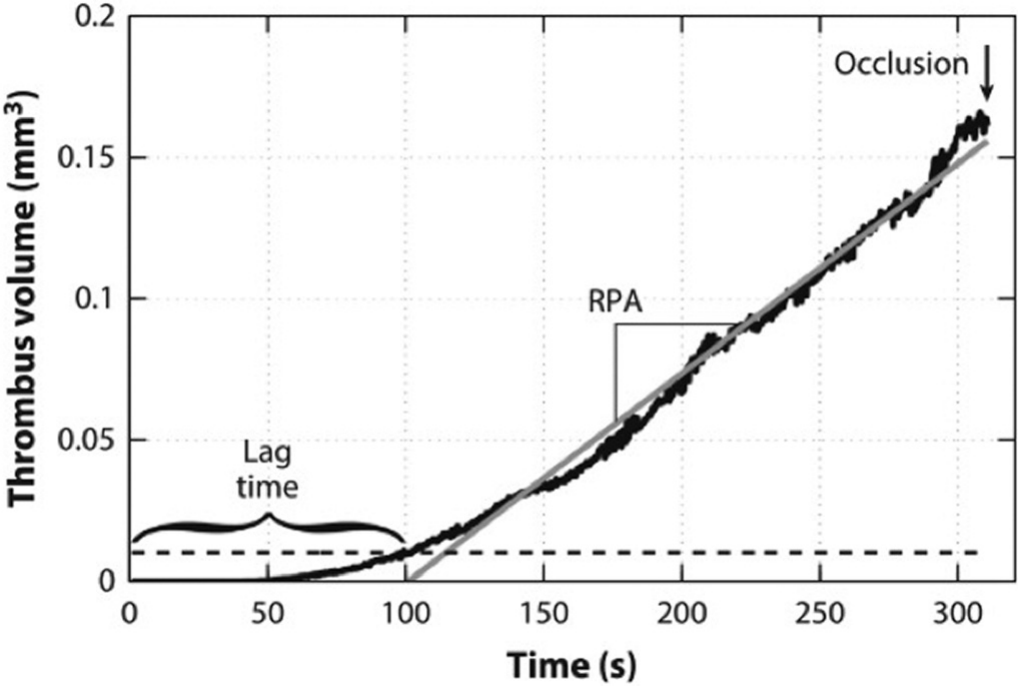
Thrombus growth occurs in three phases: phase I (lag phase), phase II (rapid platelet accumulation, RPA), and phase III (occlusion). Reproduced with permission from L. D. C. Casa and D. N. Ku, Annu. Rev. Biomed. Eng. **19**, 415–433 (2017). Copyright 2017 Annual Reviews.

Casa and Ku^25^ summarized the above mechanism of high shear thrombus formation in arteries as a 7-step process described in Table I. In the first three steps, high wall shear at the stenosis changes vWF conformation, leads to vWF absorption, and enhances platelet diffusivity to the prothrombotic subendothelial surface. Inactivated platelets adhere to the immobilized vWF through GPIbα-A1 bonds. Activation of bound platelets creates stronger bonds through integrin α_IIb_β_III_ and releases high concentrations of vWF. The additional vWF forms nets that enhance platelet binding, creating a positive-feedback system that leads to rapid platelet accumulation (RPA) and eventually occlusion (Fig. 4). Localized positive feedback loops occurring in the RPA phase could also explain the observations that thrombi have nonuniform growth with mountains and valleys protruding into the lumen^26^ and pores large enough for erythrocytes to pass through.^27^

**TABLE I.** Process of high-shear thrombus formation. Reproduced with permission from Annu. Rev. Biomed. Eng. **19**, 415–433 (2017). Copyright 2017 Annual Reviews.

1	Regions of stenosis induce high wall shear rates.
2	von Willebrand factor (vWF) attaches to the wall by adsorption onto collagen or artificial surfaces and unfolds under high-shear conditions.
3	Shear-enhanced diffusivity transports platelets to the surface and induces a high wall concentration of vWF and platelets by margination from the jostling red blood cells.
4	Circulating platelets that are not yet activated bind to surface-bound vWF.
5	Activation of adhered platelets release large amounts of vWF locally and activates integrin α_IIb_ β_3_ on the platelets for firm adhesion.
6	vWF nets form on the growing thrombus as platelets are continually captured and activated.
7	The vWF nets rapidly capture billions of platelets that accumulate to form a large thrombus, with possible vessel occlusion or embolization.

The above evidence establishes that the arterial thrombi associated with heart attacks and strokes are not formed through the traditional coagulation cascade and that vWF plays a critical role in initiating and propagating arterial thrombi formation.

## IV. vWF STRUCTURE AND TYPE IN BIOLOGICAL SYSTEMS

The unique role of vWF in arterial thrombosis is a result of its tertiary protein structure. vWF exists as long multimer chains that can reach sizes of over 20 000 kDa.^28,29^ In static conditions, a vWF multimer condenses into a globular bundle, lacking a set structure. While the monomer of vWF is 250 kDa, the functional unit of the multimer is a 500 kDa vWF dimer, approximately 120 nm long.^29,30^ These dimers form through disulfide bonds linking the C-terminals of two monomers. Dimers can associate with one another through additional disulfide bonds near their N-terminal to create long multimers ranging from 2 to 100 dimers.^31^ The largest concatemers generally have between 20 and 100 dimers, with sizes ranging from 3 to 15 μm.^31^ Within vWF, there are 12 unique domains. The most studied of these domains include the cystine knot, A1, A2, A3, C1, and D′/D3. The cystine knot region is where two monomers form a dimer. It has similar structure to other cystine knot proteins such as platelet derived growth factor (PDGF) or epithelial mucins.^32^ As the name suggests, the cystine knot domain binds the monomers with a disulfide bond.^32^ The A1 domain is known to bind the platelet receptor GPIbα, heparin, and collagen Type VI.^33–35^ The association between A1 and GPIbα is critical to the formation of a platelet rich clot, and recent research suggests that binding is dependent on shear and tension, not only for elongating vWF and exposing the A1 domains but also in transforming the A1 domain into a high affinity substrate for GPIbα.^10^ Since the A1-GPIbα bond is the first critical step in catching platelet whizzing by the stenosis in under 5 *μ*s, this bond needs to have a high $K_{on}$ rate, as previously mentioned in Ref. 9. The A2 domain controls the vWF size following excretion. As discussed below, hemodynamic forces can change the structure of A2, allowing the enzyme ADAMTS13 to cleave vWF.^36^ The A3 domain is the major binding site for collagen. A3 can bind to both triple helical and fibrillar collagen Type III, along with Type I,^37,38^ and is also regulated by the conformation of the A2 domain.^16^ The C1 domain binds integrin α_IIb_β_III_ through an Arg-Gly-Asp (RGD) motif. α_IIb_β_III_ is an integrin found on the surface of α-granules, which can bind RGD motifs on fibrinogen or vWF following platelet activation and integrin presentation.^39^ Finally, the D′D3 domains bind to and stabilize the coagulation factor FVIII. It is proposed that this link between vWF and the coagulation cascade is the reason some patients with vWF disorders have bleeding symptoms.^40^

**FIG. 5.**
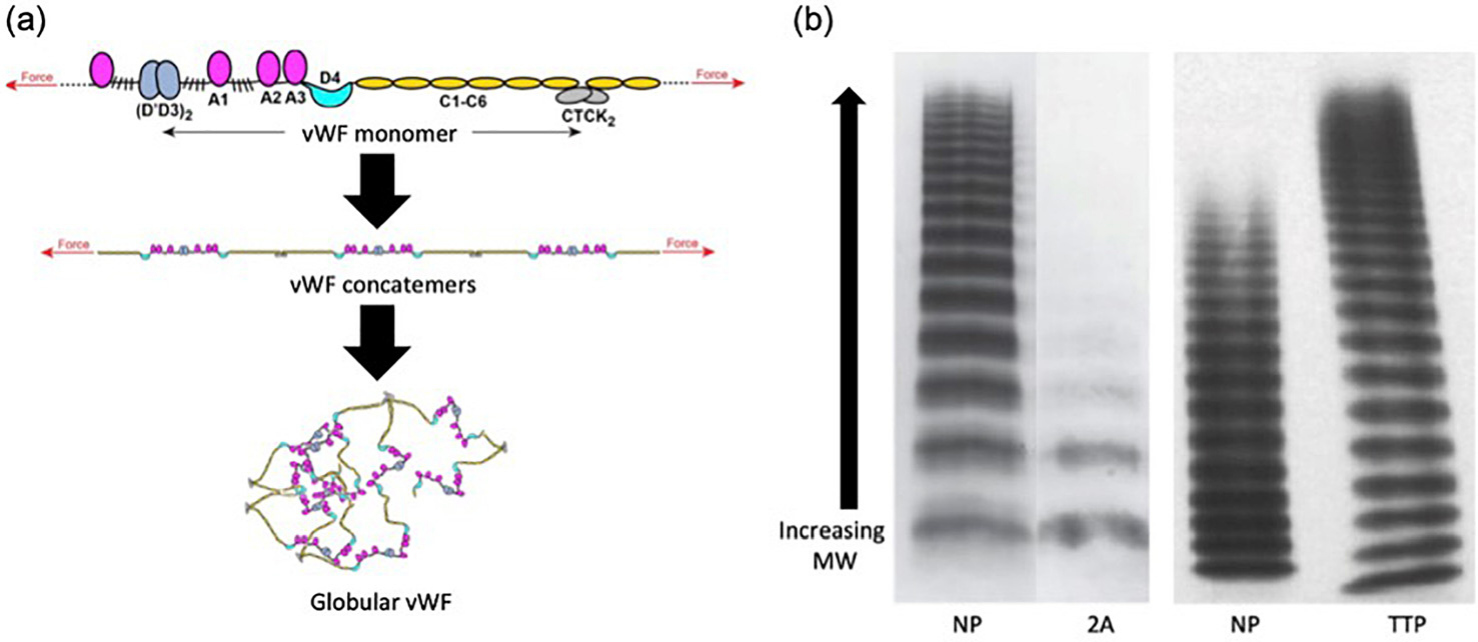
Organization and size distribution of vWF. (a) Schematic showing the organization of vWF from functional domains to globular multimers. (b) vWF multimer analysis comparing pathological conditions: Type 2A von Willebrand's disease (2A) and thrombotic thrombocytopenic purpura (TTP) to normal pooled plasma (NP). Type 2A is characterized by the lack of high molecular weight vWF, while ULvWF is more prevalent in TTP. The NP lanes for the two gels appear different because of differences in the method between the gels. Adapted with permission from T. A. Springer, “Von Willebrand factor, Jedi Knight of the bloodstream,” Blood **124**, 1412–1425, 2014. Copyright 2014 American Society of Hematology, Clearance Center, Inc.

vWF is mainly stored in two locations, within the Weibel-Palade bodies of endothelial cells and within the α-granules of platelets.^41,42^ vWF located in the plasma is secreted from endothelial cells in an ultralong state (ULvWF) at a basal level in response to factors such as thrombin or fibrin.^43,44^ The ULvWF can weigh over 20 000 kDa with up to 100 dimers. In the plasma, vWF is typically at a concentration of 10 *μ*g/ml.^45^ This roughly corresponds to ∼10 000 vWF molecules per platelet.^46^ After being secreted, ULvWF can be acted on by ADAMTS13, a plasma enzyme that cleaves between a tyrosine and methionine in the A2 domain.^47^ Studies have shown that shear stress is required to unfold the A2 domain and allow ADAMTS13 to cleave efficiently.^48^ The result of cleavage by ADAMTS13 is the creation of a spectrum of vWF lengths (Fig. 5). vWF sizes typically range from 1 to 20 dimers with the smaller sizes being more abundant.^28^ There is much evidence supporting the theory that beyond the vWF concentration, the ratio of vWF sizes plays a large role in regulating activity.^28^ Besides the vascular endothelium, vWF is also stored in the α-granules of platelets. The α-granules are secretory vesicles inside the platelets, which besides vWF also contain growth factors, fibronectin, and factor V.^49^ Following activation, the vesicles fuse with the platelet membrane, releasing the contents of the α-granules into the plasma. vWF in α-granules is shown to be at concentrations of 50-fold normal plasma levels and exists as ULvWF.^50^ This means that release from platelets can significantly increase the local concentration of ULvWF and increase the rate of platelet accumulation since vWF has been shown to be the limiting factor in the RPA phase of arterial thrombosis.^8^

When elongated by shear, vWF multimers in the plasma can associate with other elongated multimers to generate large structures.^9,24^ Intermolecular disulfide bonding leads to the creation of both large vWF fibers aligned along the direction of flow and vWF nets covering the surface of the vasculature.^24^ These fibers form at shear rates exceeding 5000 $s^{-1}$ and can reach lengths of over 100 μm.^24^ The fibers bind to multiple platelets, linking platelets that are more than one vWF multimer length apart. In vitro, these fibers have also been shown to wrap around posts, grow in length and width, and stretch due to increased fluid shear force.^51^ In addition to fibers, vWF networks formed by multiple multimers coating a surface are critical to ensure platelet attachment.^9^ As mentioned above, these vWF networks with a high surface density of A1 domains are necessary to achieve a high number of required bonds.^9^ The conformation of the nets is also critical in dictating available A1 domains. A flat, 2D vWF net exposes a 0.4 $\mu m^{2}$ area with 1115 A1 domains, while a 3D net that forms a pocket for the platelet to bind can expose a 14.1 $um^{2}$ area with 37 661 A1 domains.^9^ Thus, the α-granule released vWF and the formation of vWF nets or pockets may be critical to reach the high growth rate observed during RPA.

Deviations from the physiological vWF structure, concentration, or size distributions can lead to a family of diseases called von Willebrand's disease (vWD).^52^ Patients with vWD can suffer from bleeding pathologies such as gastrointestinal bleeding, epistaxis, menorrhagia, and bleeding from minor wounds and surgeries.^52^ However, vWD patients can also be asymptomatic, discovering their condition by accident.^53^ In contrast to the increased risk of bleeding, individuals with the highest levels of vWF have been shown to have an ∼2× increased risk of thrombotic events, such as thromboembolic stroke or acute myocardial infarction.^54^ The dual effect of vWD on bleeding and thrombosis further emphasizes the role that vWF plays alongside coagulation in balancing blood's response to abnormal blood flow, based on shear stress.

## V. PATHOLOGICAL FLUID MECHANICS OF BLOOD FLOW THROUGH STENOSIS

**FIG. 6.**
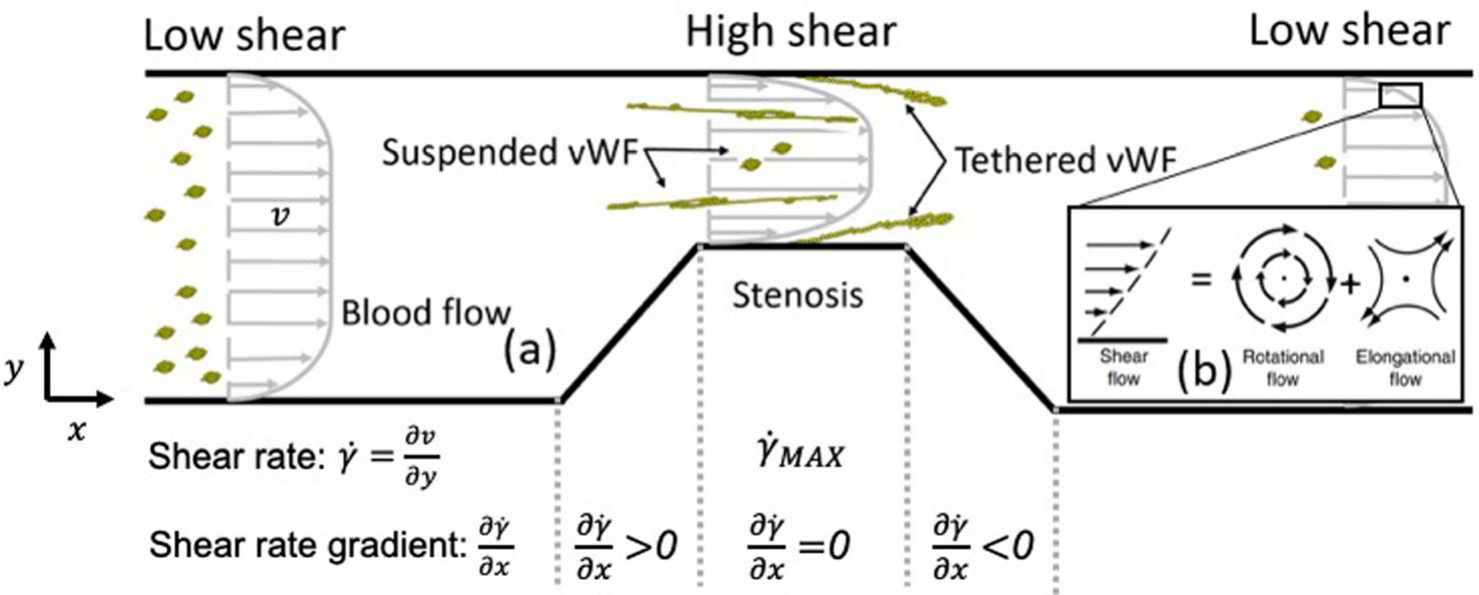
(a) Blood flow through a stenotic channel. The velocity profile is blunt due to the presence of red blood cells (RBCs). The stenosis region generates a high shear rate at the wall, leading to unfolding and potential tethering of plasma vWF near the wall. Tethered vWF gets fully stretched. (b) Shear flow can be theoretically decomposed into a rotational flow component and an elongational flow component.

Blood flow through stenotic vessels has been previously reviewed by Berger and Jou.^55^ As shown in Fig. 6(a), stenotic channels locally generate the hydrodynamic conditions needed for the growth of occlusive thrombus. As the vessel diameter constricts, velocity at the stenosis increases. The acceleration into the constriction elongates or stretches the fluid into the stenosis but shrinks it on the downstream side of the stenosis. The shear rate is the change in velocity away from the wall or velocity gradient. Wall shear rates at the stenosis can reach up to 400 000 $s^{-1}$, depending on the stenosis severity and the proximal-distal pressure drop across the stenosis.^2^ Although the shear rate is commonly reported because of the complicated viscosity of blood, it is the shear stress or viscous force per unit area that causes vWF elongation to initiate high shear thrombus formation. The shear stress is quantified by multiplying the shear rate by viscosity. A shear rate gradient refers to the change in the shear rate in the contraction and expansion regions (Fig. 6). In general, white clot forms preferentially where the shear rate and shear stress are the greatest, namely, the throat.^11–13,26,56^ In these experiments, the areas of the maximum shear rate gradient have significantly less thrombus than the throat, where no shear gradient exists. In a different series of experiments,^57,58^ when the throat is about 10 *μ*m in length, thrombus initially forms in the throat, but growth appears downstream of the throat in an expansion region or “shear microgradient.” Note that a shear gradient is also present in a converging section where “elongational flow” may be the greatest. However, this region is typically “not” where the most thrombus grows. Instead, the magnitude of the wall shear rate at the throat of the stenosis, not the presence of shear gradients, is strongly correlated with the observed clot location experimentally^59^ and clinically when the throat is on the order of 1 mm or longer.

Pulsatility of arterial flows may be another factor that affects arterial thrombosis. However, pulsatility is governed by the Womersley number, which depends strongly on the radius of the tube. Thus, microfluidics systems are poorly suited to study temporal changes in the velocity or shear rate. Full or large-scale models may be necessary to elucidate the role of pulsatility in thrombosis.

## VI. CONFORMATIONAL DYNAMICS OF SINGLE vWF IN FLOW

The conformational change of vWF in blood flow from a globular state to an elongated state is crucial to a series of arterial thrombosis relevant processes, including the elevated absorption of vWF on the collagen surface,^18^ the regulation of the vWF size through ADAMTS13,^60^ the formation of vWF nets to capture fast transit platelets,^9,24^ and so on. Given its significance in regulating arterial thrombosis, the past two decades have witnessed numerous efforts devoted to understanding the conformational dynamics of single vWF in flow. Depending on whether a vWF strand is murally tethered to the wall or freely suspended in the fluid as shown in Fig. 6(a), its elongation process features distinct characteristics in terms of unfolding shear-stress threshold and maximum multimer extension.

**FIG. 7.**
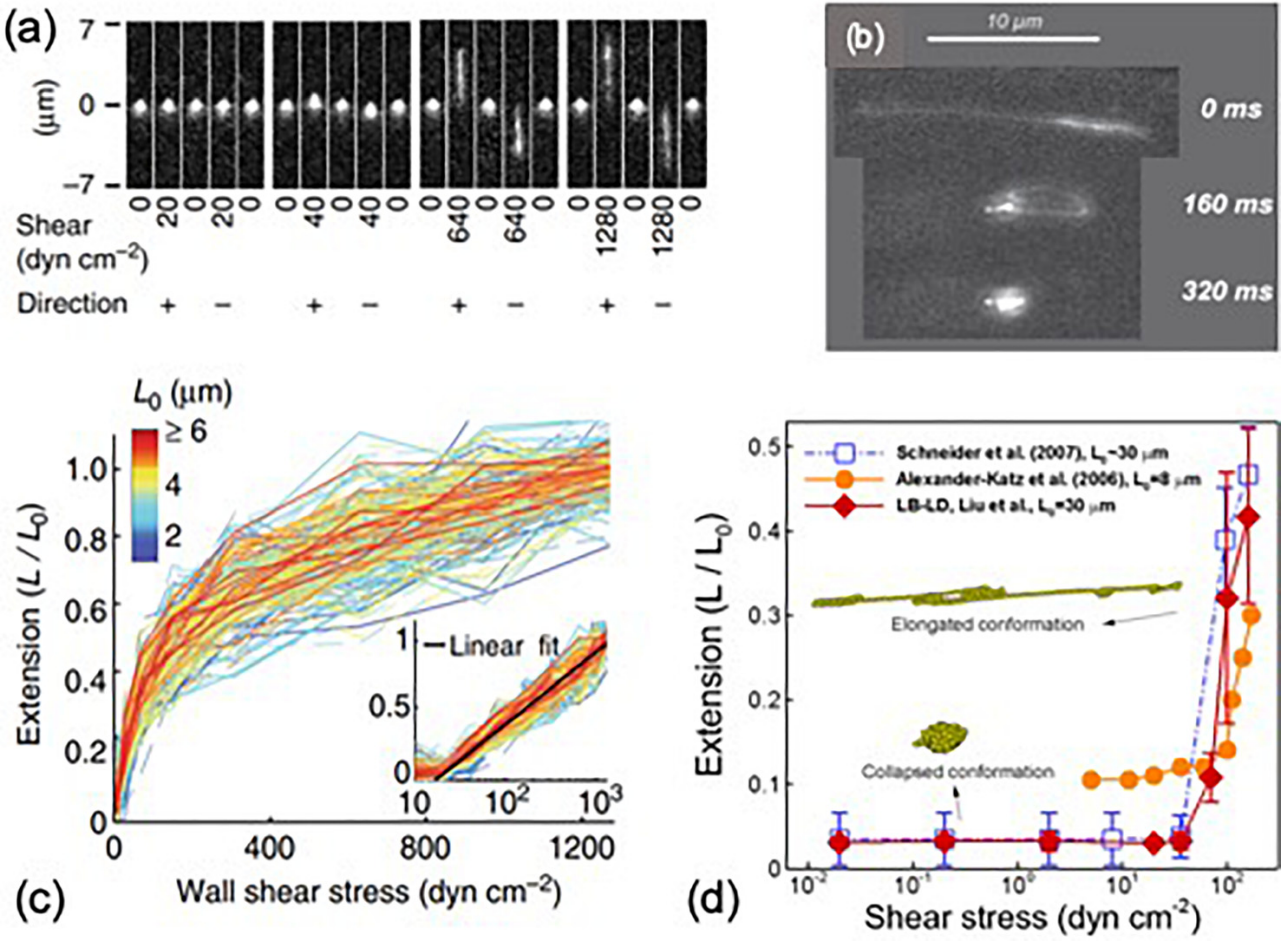
(a) Elongation of tethered vWF. Reproduced with permission from Fu *et al.*, Nat. Commun. **8**, 324–335 (2017). Copyright 2017 licensed under a Creative Commons Attribution (CC BY) license. (b) Unfolding of a freely suspended vWF in a circulating flow channel. Reproduced with permission from Schneider *et al.*, Proc. Natl. Acad. Sci. **104**, 7899–7903(2007). Copyright 2007 National Academy of Sciences. (c) Average extension of a tethered vWF under different shear stresses. Reproduced with permission from Fu *et al.*, Nat. Commun. **8**, 324–335 (2017). Copyright 2017 licensed under a Creative Commons Attribution (CC BY) license. (d) Average extension of a suspended vWF under different shear stresses. Reproduced with permission from Liu *et al.*, Int. J. Numer. Methods Fluids **91**, 228–246 (2019). Copyright 2019 John Wiley & Sons, Ltd.

Mural tethering of vWF is the prerequisite for the subsequent adhesion of platelets and anchorage of vWF-platelet aggregates under high shear. It naturally occurs *in vivo* when vWF is released from cells (such as endothelial cells or activated platelets) or when plasma vWF adheres to collagen at sites of vascular injury.^31^ Using atomic force microscopy, Siediecki *et al.*^61^ first imaged the three-dimensional tertiary structure of surface-adhered vWF subject to shear flow. They observed the elongation of vWF occurs at wall shear stresses of around 35 $\mathrm{dyn}/cm^{2}$. Fu *et al.*^10^ studied the elongation of wall-tethered vWF using microfluidics with single-molecule imaging [Fig. 7(a)], showing tethered vWF requires wall shear stresses above 20 $\mathrm{dyn}/cm^{2}$ to initiate elongation and can gradually reach 100% extension when wall shear stress increases to 1000 $\mathrm{dyn}/cm^{2}$, as shown in Fig. 7(c). Moreover, they found the longest relaxation time of vWF was weakly dependent on the multimer contour length ($∼L^{0.59}$), unlike typical polymers in fluid ($∼L^{1.5}$), which suggests there exist other mechanisms besides the Brownian effect that drives the rapid compaction of vWF strands upon decrease in fluid stresses. This finding highlights vWF as a precise biocontroller for thrombosis, allowing localized elongation and activation in response to hydrodynamic stresses in hemorrhage or thrombogenic sites and rapidly deactivation downstream.

The conformational change of freely suspended vWF [Fig. 7(b)] was not observed until the last decade by Schneider *et al.*^18^ using a circulating microfluidics system by a single molecule imaging technique. Their results reported the shear rate threshold to elongate vWF about 6000 $s^{-1}$ in plasma (corresponding to a shear stress of ∼100 dyn/$cm^{2}$), which is higher than the unfolding shear-stress threshold for tethered vWF (20–35 dyn/$cm^{2}$).^10,61^ Another big difference is that tethered vWF can be up to 100% elongated, while the average extension ($L/L_{0}$) of suspended vWF, shown in Fig. 7(d), remains below 50%^18,62^ due to tumbling motion induced by the rotational flow component of shear flow [Fig. 6(b)].

Mural tethering substantially reduces the vWF unfolding shear-stress threshold and can lead to 100% extension and formation of vWF nets that can capture fast transiting platelets under elevated shear stresses.^9,24,63^ Although studies focused on lag time phenomena^7,18^ show that soluble vWF plays a critical role in the formation of platelet aggregates at high shear rates (6000–20 000 $s^{-1}$), much effort should be devoted to directly addressing the role of tethered vWF and formation of vWF nets in thrombosis to understand the biomolecular mechanism of explosive platelet accumulation and occlusion.

## VII. COMPUTATIONAL MODELING OF HIGH SHEAR THROMBOSIS

Computational modeling of high shear thrombus formation is both fundamentally pivotal in understanding the governing mechanism of arterial thrombosis and practically beneficial in predicting the location and risk of thrombosis in human and blood contacting devices. Simulating such fluidized clotting processes requires resolving a variety of biophysical, biochemical, and computational complexities, including fluid-solid interactions, transport, and deposition of platelets and biomolecules into thrombus, ligand-receptor based binding between active components, large spatiotemporal-scale discrepancy, and so forth, which make it a grand challenge for computational modeling.

**FIG. 8.**
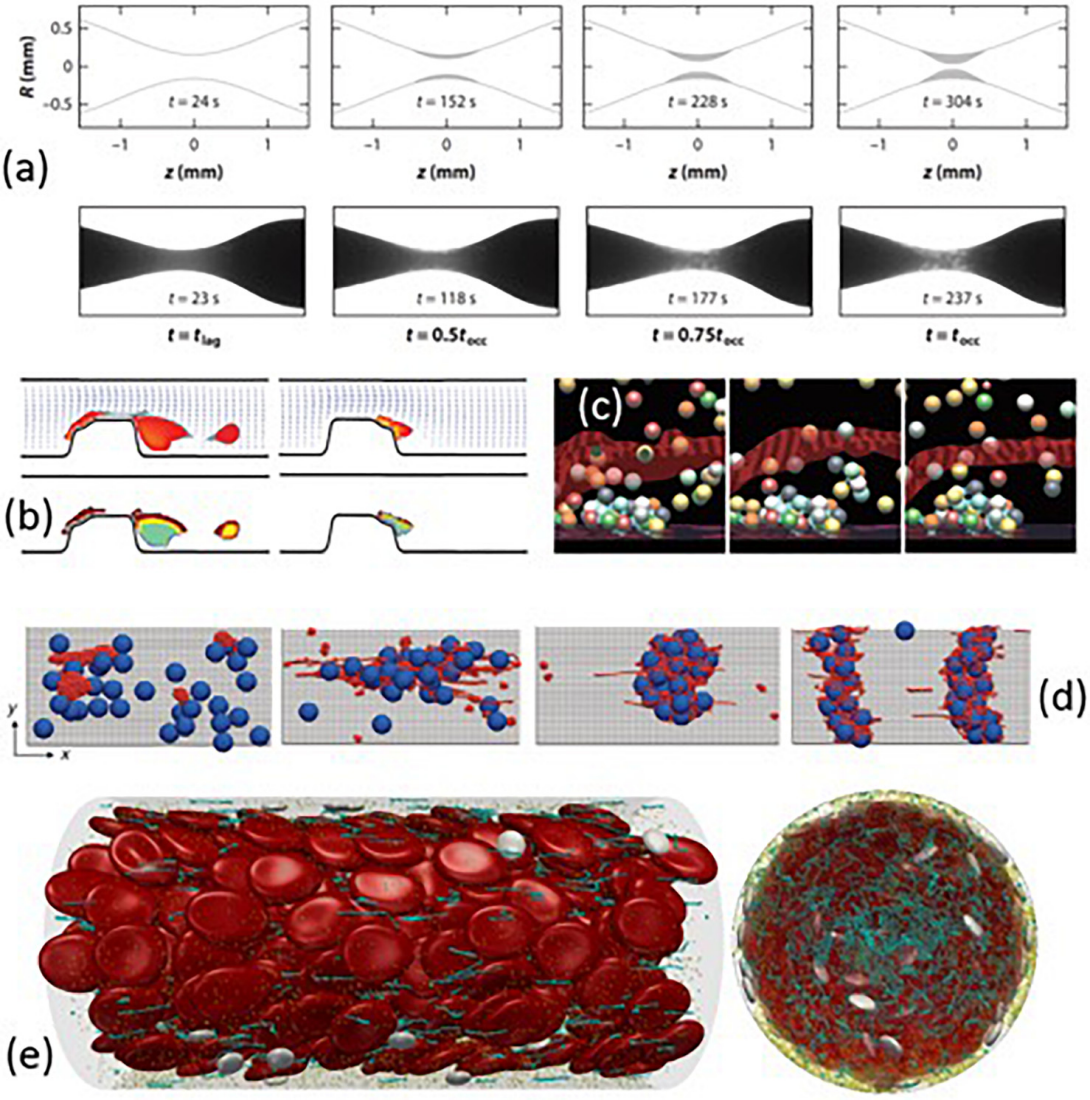
(a) High shear thrombosis formation over time: The comparison between the phenomenological model and *in vitro* experiment. Reprinted with permission from Mehrabadi *et al.*, “A predictive model of high shear thrombus growth,” Ann. Biomed. Eng. **44**, 2339–2350. Copyright 2016 Springer Nature. (b) Formation and embolization of thrombus near a stenosis. Reproduced with permission from A. L. Fogelson and R. D. Guy, Comput. Methods Appl. Mech. Eng. **197**, 2087–2104 (2008). Copyright 2008 Elsevier B.V. (c) Platelet activation and adhesion at a thrombogenic surface. Reproduced with permission from A. L. Fogelson and R. D. Guy, Comput. Methods Appl. Mech. Eng. **197**, 2087–2104 (2008). Copyright 2008 Elsevier B.V. (d) Shear-induced colloid-polymer aggregate formation at various shear rates. Reprinted with permission from Chen *et al.*, “Blood-clotting-inspired reversible polymer-colloid composite assembly in flow,” Nat. Commun. **4**, 1333. Copyright 2013 Springer Nature. (e) Multiscale modeling of complex blood flow through a microvessel. Reproduced with permission from Liu *et al.,* Int. J. Numer. Methods Fluids **91**, 228–246 (2019). Copyright 2019 John Wiley & Sons, Ltd.

Instead of modeling all constituents of high shear thrombosis, phenomenological models^12,64^ predict the occlusive thrombus formation based on the experimentally determined thrombosis lag time and the rates of rapid platelet accumulation as functions of shear rate. As a result, these models can predict large-scale, long-time thrombus formation and occlusion, as shown in Fig. 8(a). Practically, such phenomenological models are useful for evaluating the thrombogenic risks and predicting the occlusion time in diseased arteries or blood contacting devices.^12^ However, these models based on experimental measurements lack the ability to provide detailed biomechanistic insights into high shear thrombosis from first principles.

Continuum models^65–67^ treat thrombus growth as a multiphase problem by calculating the platelet number concentration based on a set of convection-diffusion-reaction equations [e.g., Fig. 8(b)]. These models can be efficiently used to predict thrombus formation in macroscopic regimes and investigate the geometric and hemodynamic sensitivities of the high shear thrombosis. However, the applicability of these models highly relies on accurate constitutive relations such as the drift diffusivity of platelets and molecules^68,69^ and adhesion/reaction rates of platelets.^70–72^ Also, these approaches typically neglect the discrete nature of thrombus formation and do not consider the anisotropy and heterogeneity of the clot microstructure. Another limitation is that the scale of continuum models cannot be used to study the ligand-receptor based platelet-platelet or vWF-platelet interaction for understanding the mechanism of high shear thrombus formation.

Discrete particle-based models explicitly simulate platelet dynamics and binding^66,73–76^ and interaction with red blood cells and walls^77–80^ with substantial computational expense; see Fig. 8(c), for example. Compared to continuum models, the particle-based approach can predict the thrombus structure down to cellular scales. However, most of the efforts have directly considered platelet-platelet adhesion without including vWF. As a result, these models cannot explain some critical mechanisms of high shear thrombosis such as the shear-dependence of the thrombus accumulation rate;^12,25,64^ instead, this type of information is often incorporated into the platelet adhesion model to render shear-dependent platelet accumulation.^73^

Recently, several multiscale particle-based approaches^15,63,81,82^ have been developed to directly incorporate nanoscale vWF molecules with microscale cells in order to tackle thrombosis. Chen *et al.*^81^ simulated shear-induced polymer-colloid aggregation mimicking vWF-platelet aggregates using a coupled lattice-Boltzmann (LB) and Brownian dynamics method. Their results captured the shear-dependent microstructures of the aggregates, as shown in Fig. 8(d)—the low shear rate induces streamwise and loose aggregates; the intermediate shear rate leads to compact and circular aggregates; the high shear rate leads to cross-stream log-shape aggregates. Rack *et al.*^15^ studied vWF margination in tubular blood using a dissipative particle dynamics (DPD) method, showing the globular conformation of vWF under low shear is important for its margination toward the wall and enables its further tethering and unfolding at the wall. Liu *et al.*^63,82^ developed a two-way coupled LB and Langevin-dynamics (LB-LD) approach to simulate the multiscale blood constituents in flow. The LB-LD approach can seamlessly simulate the dynamics and interactions of vWF, and platelets and red blood cells flow through microvessels under physiological and pathological conditions, as shown in Fig. 8(e). The LB-LD approach was recently used to study the effect of charged nanoparticles (CNPs) on the inhibition of thrombosis,^83^ where CNPs were found to induce recoil of vWF under high shear due to the electrostatic interactions between CNP and vWF.^83^

Substantial effort has been made to simulate high shear thrombosis over the past two decades, and yet, a predictive and fundamentally informative model that can tackle occlusive thrombosis remains to be developed. Given the crucial role of vWF in high shear thrombosis, incorporating vWF or its effects into the model poses an opportunity as well as a challenge toward the development of first-principles based models that can potentially lead to better biomechanistic understanding of the occlusive thrombus formation and direct the design of experimental work. The development of such multidisciplinary models also calls for closer communication among researchers in biology, computer science, physics, chemistry, and medical research areas.

## VIII. POTENTIAL vWF TARGETING THERAPIES

Given the critical role of vWF in arterial thrombosis, it is a natural potential target for preventing the RPA associated with ischemic events like heart attacks and strokes. While traditional antithrombotic agents typically focus on preventing platelet activation or inactivating factors in the coagulation cascade, they are associated with an increased risk of bleeding and can therefore be dangerous if administered inappropriately.^84^ A vWF-targeted therapy has the potential to overcome this current shortcoming of these drugs and act orthogonally without affecting many other platelet functions.

As the physiological enzyme responsible for cleaving vWF, ADAMTS13 is a natural first choice for targeting vWF. It has been shown that low levels of ADAMTS13 are associated with increased risks of arterial thrombosis.^85^ However, the results from experiments attempting to prevent clot formation or lyse clots after formation have been mixed. In one experiment, ADAMTS13 failed to cause vWF strings to dissociate; but it was shown to reduce the size of tissue plasminogen activator (tPA) resistant clots in mice.^51,86^ Further work is needed to determine the practicality of using ADAMTS13 for preventing and treating arterial thrombosis.

Another proposed method of targeting vWF comes from the observation that patients on extracorporeal membrane oxygenator (ECMO) circuits can cause the temporary loss of high molecular weight vWF multimers, a sort of acquired Type IIA vWD.^87^ This is also seen in patients with severe valve stenoses and other types of pumps.^88^ A hypothesis is that vWF shortening is caused by exposing the blood to high shears since the blood in pumps can experience shears between 500 and 2500 dyn/cm^2^.^89^ The loss of long multimers is correlated with increased bleeding, and treatment involves delivering both vWF and FVIII together.^90,91^ While the shear stresses are high enough to cause platelet activation, devices like extracorporeal circuits could be used to decrease the size of an individual's vWF.

A new method of targeting vWF to prevent arterial thrombosis involves the use of charged nanoparticles (CNPs). Computational modeling of vWF predicted that while vWF naturally transitions from globular to partially elongated forms under flow, the addition of negatively charged particles could cause vWF to favor a globular state, with very few A1 domains exposed for binding.^83^ The A1 domains of vWF have a positive charge, which helps A1 electrostatically attract the negatively charged GPIbα receptors.^10^ Testing this prediction, it has recently been shown computationally that the addition of negatively charged nanoparticles may reduce the rate of platelet accumulation.^83^ Recently, we have shown that CNPs have indeed prolonged occlusion times in whole blood.^83^ More research is needed to explain this behavior and optimize efficacy *in vivo*.

Finally, there is the potential to target specific domains in vWF with antagonists that prevent platelet binding. This could potentially overcome the bleeding complications involved with platelet targeting molecules. An example would be antibodies targeting the A1 domain, such as ALX-0081 (Caplacizumab).^92^ In a baboon modified Folts model, monoclonal antibodies against A1 were shown to be as effective as antiplatelets in preventing clot formation and led to less bleeding from a standardized wound.^92^ Another promising vWF-targeted therapy is the aptamers developed in Ref. 93. These aptamers have been designed to have a high affinity for vWF, and as a result, they observe a significant reduction in platelet accumulation. Interestingly, the aptamers are also able to recanalize occluded vessels that had stabilized for 20 min. While the aptamer did cause an increase in bleeding times, the complementary antidote was able to reverse the increase effect on bleeding within 5 min. These findings support specific vWF antagonists as a viable way to prevent and treat arterial thrombosis.

## IX. FUTURE DIRECTIONS

Over the past two decades, substantial progress has been made in understanding the high-shear thrombosis and how vWF plays a pivotal role throughout the full spectrum of thrombus formation. However, there are still big unanswered questions surrounding vWF. Listed below are some of the topics that future research can aim to answer.

What aspects of vWF function play a different role in arterial thrombosis compared to hemostasis? While the role of vWF in unfolding and binding platelets in high shear conditions has been characterized, its role in hemostasis is less clear and compounded by the relationship between vWF and FVIII. This would be important to consider when treating patients with vWD, who sometimes present with excessive bleeding. Another challenge in answering this question is that “bleeding” is a broad term and the conditions during bleeding, which lead to hemostasis, are poorly defined. A bleed from an arterial laceration is quite different from a mucosal bleed or bruising from capillaries. Widely different shear stresses and resistances are involved, the blood contacts different surfaces, and the bleeding can be directly out of the end of the lumen of a high-pressure artery or through the wall of a venule. Multiple groups are working on designing better microfluidic systems for small-scale *in vitro* testing.^94–96^ More research to better classify types of bleeding and develop standardized models of each type is crucial for untangling the mechanisms in hemostasis.

How do the vWF concentration, size, and origin interplay affect the blood's ability to create occlusive clots *in vivo*? One of the most common assays for vWF is the vWF antigen test, but this test fails to provide any insight into multimer size or functionality. Western blots are able to distinguish abnormalities in multimer size, but they are not simple or common and have difficulty in identifying the larger sizes of vWF. Many vWF studies focus on plasma vWF, but recent research suggests the vWF released from platelets plays an important role in increasing local vWF concentrations to speed clot formation and clot stabilization.^8,9,97^ Further research into the molecular mechanisms for packaging vWF into α-granules and triggering the release of α-granules could lead to therapeutics that reduce the amount of vWF released by platelets without globally inhibiting platelet activation.

Can we balance thrombosis and bleeding risk by controlling the vWF length? ULvWF strands increase the risk of a major adverse cardiac event (MACE) occurring, but the substantial reduction in the vWF size can cause acquired vWD and bleeding. Studies by Fu *et al.*^10^ have shown that the length can affect folding kinetics and drag forces felt by a tethered vWF molecule. Along with the observation that patients on external pumps experience a reduction in the vWF length, this suggests that the vWF length might be a variable that we can alter to lower the risk of MACE. Computational models can combine these observations to model if shortening average vWF lengths will increase the necessary shear rate threshold and lower the rate of thrombus growth. Then, the question becomes can we verify the predictions with experiments and how do we properly adjust the balance of the vWF length to reduce the risk of mortality for patients?

Can we develop simple small molecule drugs to antagonistically target vWF? The majority of current antithrombotic agents target platelets and platelet activation, and it is not surprising that these drugs also affect coagulation and hemostasis. vWF targeted drugs can potentially overcome this limitation. Current work with antibodies and CNPs supports the validity of these treatments but comes with a large price tag. The development of small molecule drugs acting on vWF could provide a more efficacious way to prevent MACE while not causing life-threatening bleeding.

The ultimate goal of this research is developing therapies to prevent and treat arterial thrombosis. As the field answers these and other questions, we will continue to build our understanding of the mechanisms of thrombosis and hemostasis and the difference between them to better design and optimize novel diagnostics and treatments.

## X. SUMMARY

Shear stress from blood flow causes vWF to transition from a globular form to an elongated form. This change in the tertiary structure of vWF allows it to dynamically respond to local pathological hemodynamic conditions. Elongated vWF self-associates into nets almost instantaneously and binds to both collagen and platelets. White clots can form to occlude major arteries, leading to heart attack and stroke. The measurement of detailed mechanical of vWF may allow us to model its behavior in flow and use those models to predict clot formation. As the crucial protein for arterial thrombosis, vWF targeting may provide a method for selectively preventing stenotic occlusion without concomitant bleeding.

As we move to develop future treatments, we may extend beyond describing the behavior of vWF to determine the factors that control its behavior. A more complete mechanism of occlusive arterial thrombosis, thrombolysis, and hemostasis may reveal possible new interventions. Biomechanistic, computational models that predict the effects of these many interventions may create a list of novel promising treatments. vWF plays a key role in preventing and treating life-threatening heart attacks and strokes.
